# Cartilaginous bending spring for preventing tympanic membrane graft medialisation in anterior or subtotal tympanic membrane perforations—how I do it

**DOI:** 10.1007/s00405-021-06814-5

**Published:** 2021-04-21

**Authors:** Konstantinos Mantsopoulos, Heinrich Iro, Joachim Hornung

**Affiliations:** Department of Otorhinolaryngology, Head and Neck Surgery, Friedrich-Alexander University Erlangen-Nuremberg (FAU), Waldstrasse 1, 91054 Erlangen, Germany

**Keywords:** Tympanic membrane, Tympanic perforation, Cartilage, Graft medialisation

## Abstract

**Background:**

The reconstruction of anterior or subtotal tympanic membrane perforations is critical due to the risk of anterior graft medialisation and retraction or recurrent perforation.

**Method:**

After reconstruction of the tympanic membrane by means of grafting, a rectangular cartilage strut (length 6 mm, breadth 2 mm, thickness 0.1 mm) is prepared using a cartilage knife and scalpel. This strut graft is placed between the cartilage graft and the promontory in the anterior inferior part of the middle ear cavity.

**Conclusion:**

Our experience shows that using a *U*-shaped cartilage strut to sustain the tympanic reconstruction effectively prevents the medialisation of the graft and recurrent perforations.

**Supplementary Information:**

The online version contains supplementary material available at 10.1007/s00405-021-06814-5.

## Relevant surgical anatomy

From an anatomical point of view, the anterior bony overhang as well as a narrow anterior tympanomeatal angle preclude complete visualisation of the tympanic membrane (TM) on otoscopy. In addition, the TM lies obliquely, from lateral superior posterior to medial inferior anterior, forming an angle of about 45° to the sagittal plane. The handle of the malleus draws the TM slightly inwardly, forming a funnel shape. Elevation of the posterior tympanomeatal flap and denuding of the malleus handle eliminate the funnel shape of the TM and create a gap between the still medially directed malleus handle and the eardrum. The direction of the malleus handle towards the promontory is responsible for inadequate graft support and a higher risk of graft failure in cases with “underlay” reconstruction of the anterior part of the TM. Furthermore, the low vascularisation in the anterior TM (compared to posterior) is disadvantageous with respect to wound healing [1]. The dysfunction of the auditory tube with the resulting critical pneumatisation of the middle ear in such cases increases the risk of graft medialisation and recurrent anterior TM perforation.

In cases of anterior or subtotal perforation many authors propagate the more challenging overlay technique, whose disadvantages, however, include graft lateralisation, anterior blunting, delayed healing, the formation of cholesteatoma and canal stenosis [2]. Remarkably, a large variety of techniques have been recommended for this demanding entity: the anterior tab flap technique [3], the anterior “mucosal pocket” myringoplasty [4], the three-point fix technique [5] or sandwich graft and window shade tympanoplasty [6], or simply middle ear packing with dry Gelfoam. In this article, we would like to introduce a *U*-shaped cartilage strut for anterior support of the tympanic reconstruction. The superior stability of cartilage in comparison to the sole use of perichondrium or temporalis fascia in the demanding postoperative environment of a chronic ear makes it an advantageous material for surgical reconstruction of the middle ear anatomy. A further advantage of this graft over other techniques is that it takes the natural middle ear anatomy into account and does not require surgical manipulations in critical structures with the risk of iatrogenic complications, e.g. in the anterior tympanomeatal angle (risk of blunting).

## Description of the surgical technique

**Fig.1:**
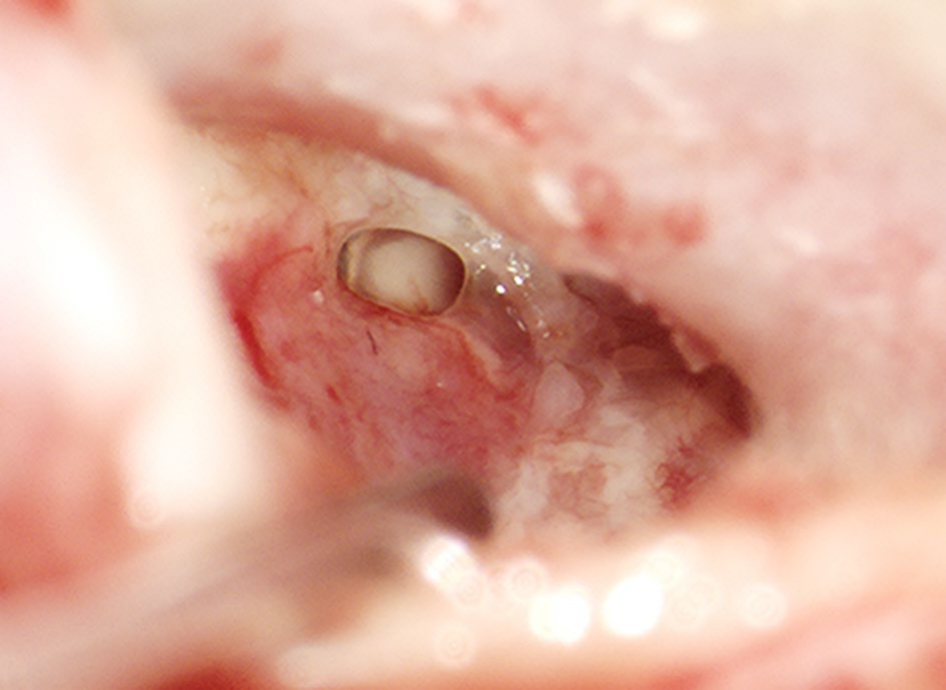
Recurrent perforation in the anterior-inferior quadrant of the right tympanic membrane

**Fig. 2:**
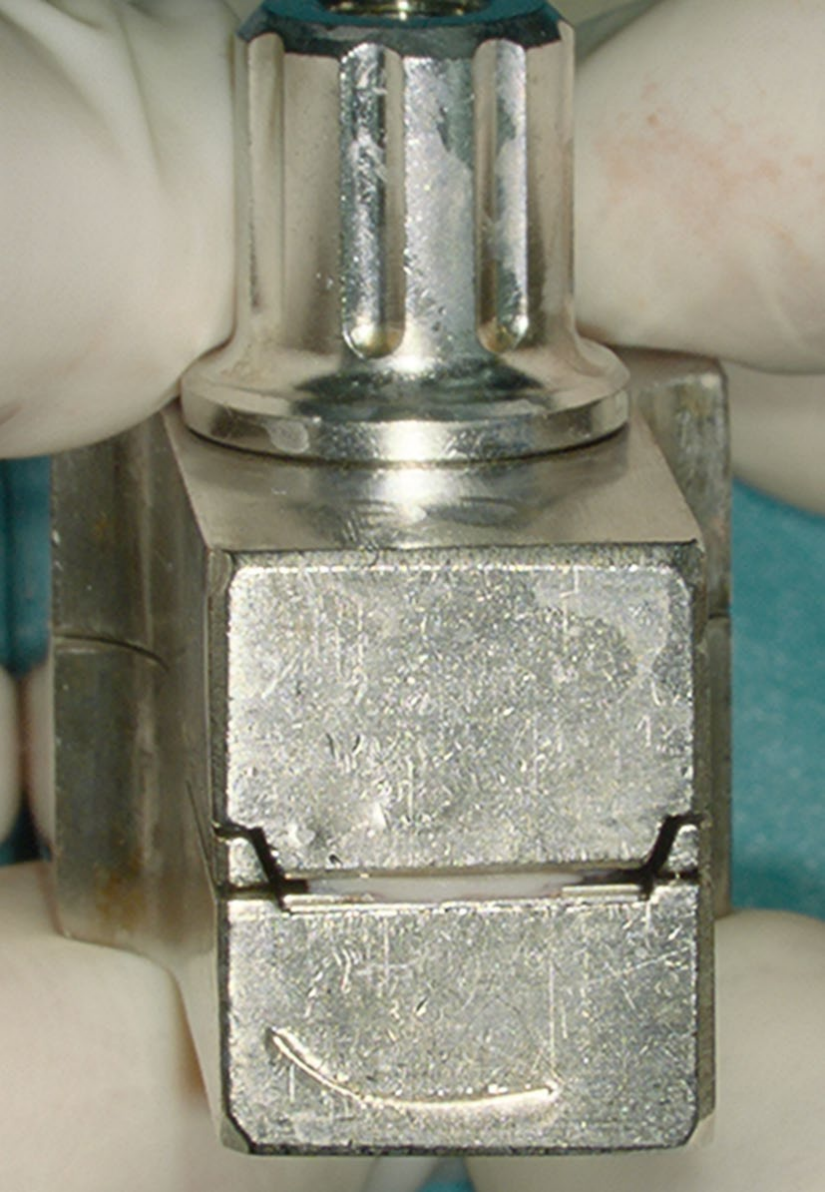
Apple further piece of cartilage is trimmed to a slice with a thickness of something more than 0.1 mm using a cartilage knife (KURZ Precise Cartilage Knife, BESS Medizintechnik Gmbh, Berlin, Germany)

**Fig. 3:**
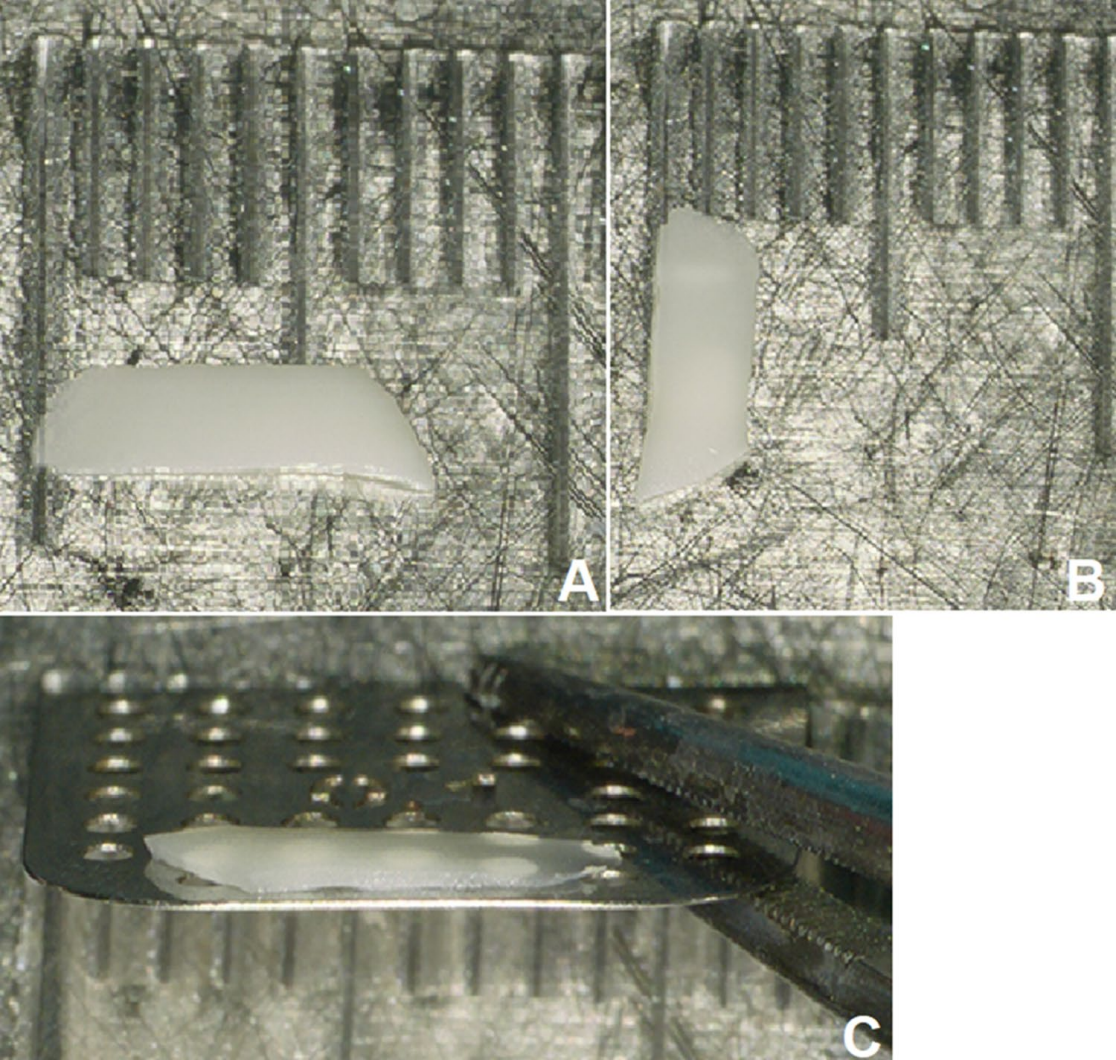
This piece of cartilage is cut into a rectangular shape (**a** length 6 mm, **b** breadth 2 mm) with a No. 15 scalpel and is a bit thicker than a 0.1 mm distance plate **c**

After the induction of general anaesthesia and orotracheal intubation, a local anaesthetic (ultracain 2% with adrenalin 1:200,000) is infiltrated into a subperiostal plane. An endaural incision is made in the area between the tragus and the crus of helix, and a self-retaining retractor is placed. The soft tissue is elevated to the level of the bony ear canal and the posterior vascular tympanomeatal flap is elevated along with the posterior annulus to enter the middle ear space, evaluate the ossicular chain status and exclude the presence of any pathology. In cases of anterior or subtotal perforation (Fig. 1), it is frequently difficult to inspect the anterior edge of the perforation. In these cases, a horizontal skin incision is made on the anterior wall of the ear canal parallel to and as far as 20 mm lateral to the anterior fibrous annulus. The bone of the anterior wall is drilled with a diamond burr until the anterior margin of the perforation can be sufficiently seen. The edges of the perforation are freshened with a 1.5-mm hook to ensure complete de-epithelialisation of the medial surface of the tympanic membrane remnant and promote the future migration of epithelium onto the lateral surface of the graft. The malleus handle and the umbo are denuded. Myringosclerotic plaques are removed. A (tragal or conchal) cartilage graft as well as a perichondrium graft are placed as an underlay (to the tympanic membrane remnant) and an overlay (to the handle of the malleus). Following this, a further piece of cartilage is trimmed to a slice with a thickness of something more than 0.1 mm using a cartilage knife (KURZ Precise Cartilage Knife, BESS Medizintechnik Gmbh, Berlin, Germany) (Fig. 2) and cut into a rectangular shape (length: 6 mm, breadth: 2 mm) with a No. 15 scalpel (Fig. 3). These dimensions as well as the straight (not rolled-up) form of the strut secure the ideal combination of graft rigidity and elasticity. The length of the strut can be adjusted according to the surface of TM grafting needing to be sustained. This cartilage graft is subsequently placed between the reconstructed TM and the promontory, taking the form of a *U*-shaped bending spring (with its opening facing outwards and slightly upwards) in the anterior inferior part of the middle ear cleft (Video). It is important that this cartilaginous bending spring remains in contact with the cartilaginous (and not the perichondrial) tympanic graft and is placed so far anteriorly that it reaches the bony anterior wall of the middle ear and the tympanic sulcus. The tympanomeatal flap is brought back into place (Video), silicone strips stabilise the skin grafts in contact with the underlying bone and Gelfoam is used to pack the ear canal. Finally, the endaural incision is closed.

Inspection of the middle ear, e.g. by second-look surgical procedures has verified the integration of cartilage bending spring in the environment of the middle ear. We could detect a complete epithelisation with a normal mucosal lining over the cartilage strut, similar to the epithelisation of the medial surface of the cartilage grafting of the eardrum. Even in cases with a high risk of a postoperative adhesive process (e.g. reduced middle ear aeration), positioning the strut in the anteroinferior part of the middle ear leaves enough space for placing silicone sheets to prevent adhesions between the eardrum and the promontory.

## Indications

Anterior or subtotal perforations independent of the underlying pathology.

## Limitations

Sufficient anterior support is achieved through a cartilage bending spring with a thickness of somewhat more than 0.1 mm exerting its medial pressure to a cartilage graft of the eardrum, which is usually not thicker than 0.3 mm. Either an excessively thick eardrum cartilage or an overly thin cartilage strut (≤ 0.1 mm) bears the risk of loss of support and medialisation. On the other hand, an increased thickness of the strut graft leads to an increased rigidity, with loss of the U-shape, strut displacement and resulting lateralisation of the reconstructed eardrum. Reasonably, in such cases, increased tension of the tympanic membrane or a recurrent perforation could lead to conductive hearing loss. Experience in otologic surgery as well as the use of special cartilage knives for cutting slices of cartilage with a predefined thickness are essential for the described technique.

## How to avoid complications

The exact measurement and preparation of the U-shaped cartilage strut are of major importance. Increasing experience in otologic surgery, strict adherence to the recommended dimensions (especially the thickness) of the strut as well as proper positioning so far anteriorly that it reaches the bony anterior wall of the middle ear and the tympanic sulcus are crucial for avoiding displacement. With increasing experience, the surgeon is able to identify intraoperatively whether the strut will remain in situ or become displaced in the course of time.

## Key points

1. The reconstruction of anterior or subtotal tympanic perforations is critical due to inadequate anterior graft support.
2. This contribution suggests using a cartilaginous bending spring to prevent tympanic membrane graft medialisation.
3. After graft reconstruction of the TM, a further piece of cartilage is trimmed to a slice with a thickness > 0.1 mm using a cartilage knife.
4. This cartilage slice is cut into a rectangular shape (length 6 mm, breadth 2 mm) with a No. 15 scalpel.
5. The resulting cartilage strut is placed between the tympanic membrane and the promontory, forming a U-shaped bending spring (with its opening facing superior posterior) in the anterior-inferior part of the middle ear cleft.
6. If needed, two separate cartilage struts can be used as U-shaped bending springs.
7. The cartilaginous bending spring should remain in contact with the cartilaginous graft and reach the bone of the anterior wall of the middle ear and the tympanic sulcus.
8. The exact measurement and preparation of the cartilage strut is of major importance.
9. Increased thickness of the strut leads to increased rigidity, with loss of the U-shape and a risk of displacement.
10. A diminished thickness of the cartilage strut would lead to an unsatisfactory support of the tympanic graft.

## Supplementary Information

Below is the link to the electronic supplementary material.

Supplementary file1 (MP4 59905 KB)
